# A mixed community of actinomycetes produce multiple antibiotics for the fungus farming ant *Acromyrmex octospinosus*

**DOI:** 10.1186/1741-7007-8-109

**Published:** 2010-08-26

**Authors:** Jörg Barke, Ryan F Seipke, Sabine Grüschow, Darren Heavens, Nizar Drou, Mervyn J Bibb, Rebecca JM Goss, Douglas W Yu, Matthew I Hutchings

**Affiliations:** 1 School of Biological Sciences, University of East Anglia, Norwich, Norwich Research Park, NR4 7TJ, UK; 2 School of Chemistry, University of East Anglia, Norwich, Norwich Research Park, NR4 7TJ, UK; 3 The Genome Analysis Centre, Norwich, Norwich Research Park, NR4 7UH, UK; 4 Department of Molecular Microbiology, John Innes Centre, Norwich, Norwich Research Park, NR4 7UH, UK; 5 State Key Laboratory of Genetic Resources, and Evolution, Ecology, Conservation and Environment Center (ECEC), Kunming Institute of Zoology, Chinese Academy of Sciences, Kunming, Yunnan 650223, China; 6 School of Medicine, Health Policy and Practice, University of East Anglia, Norwich, Norwich Research Park, NR4 7TJ, UK

## Abstract

**Background:**

Attine ants live in an intensely studied tripartite mutualism with the fungus *Leucoagaricus gongylophorus*, which provides food to the ants, and with antibiotic-producing actinomycete bacteria. One hypothesis suggests that bacteria from the genus *Pseudonocardia *are the sole, co-evolved mutualists of attine ants and are transmitted vertically by the queens. A recent study identified a *Pseudonocardia*-produced antifungal, named dentigerumycin, associated with the lower attine *Apterostigma dentigerum *consistent with the idea that co-evolved *Pseudonocardia *make novel antibiotics. An alternative possibility is that attine ants sample actinomycete bacteria from the soil, selecting and maintaining those species that make useful antibiotics. Consistent with this idea, a *Streptomyces *species associated with the higher attine *Acromyrmex octospinosus *was recently shown to produce the well-known antifungal candicidin. Candicidin production is widespread in environmental isolates of *Streptomyces*, so this could either be an environmental contaminant or evidence of recruitment of useful actinomycetes from the environment. It should be noted that the two possibilities for actinomycete acquisition are not necessarily mutually exclusive.

**Results:**

In order to test these possibilities we isolated bacteria from a geographically distinct population of *A. octospinosus *and identified a candicidin-producing *Streptomyces *species, which suggests that they are common mutualists of attine ants, most probably recruited from the environment. We also identified a *Pseudonocardia *species in the same ant colony that produces an unusual polyene antifungal, providing evidence for co-evolution of *Pseudonocardia *with *A. octospinosus*.

**Conclusions:**

Our results show that a combination of co-evolution and environmental sampling results in the diversity of actinomycete symbionts and antibiotics associated with attine ants.

## Background

Fungiculture in the insect world is practised by ants, termites, beetles and gall midges [1]. The best-characterized examples are the attine ants, which are endemic to South and Central America and to the southern USA. The ancestor of these ants evolved the ability to cultivate fungus as a food source around 50 million years ago, leading to the monophyletic tribe Attini, which number 12 genera with more than 230 species. The genera *Acromyrmex *and *Atta *(40 species) evolved 8-12 million years ago and form a branch of the higher attines, also known as leaf-cutting ants, which are characterized by large colonies of up to several million individuals [2]. Like the other leaf-cutting ants, the well-studied species *Acromyrmex octospinosus *forms a mutualism with a single basidiomycete fungus (Agaricales: Lepiotaceae: Leucocoprineae) *Leucoagaricus gongylophorus *in which they exchange food as well as protection and transport services [3].

The mutualistic fungal garden can be parasitized by a variety of other fungi [4] but the major pathogen of leaf-cutting ant fungal gardens is a necrotrophic fungus (Ascomycota: anamorphic Hypocreales) in the genus *Escovopsis *[5]. Around 25% of the gardens in Panamanian ant colonies contain *Escovopsis *which feed on the fungal cultivar and can destroy fungal gardens, leading to the collapse of the colony [6].

There is evidence that the fungal cultivar produces antibiotics in order to defend itself [7-9] and the ant workers also defend their fungal gardens through a combination of grooming and weeding [8], production of their own antimicrobials through metapleural gland secretions [10] and the application of weedkillers. These weedkillers are natural product antimicrobials produced by symbiotic actinomycete bacteria [7,11-13]. A long-standing theory suggests that bacteria from the genus *Pseudonocardia *co-evolved with the ants and are transmitted vertically by the gynes (reproductive females) along with the fungal cultivar. However, more recently, evidence has emerged that suggests attine ants are also associated with bacteria from the actinomycete genera *Streptomyces *and *Amycolatopsis *and that antibiotic-producing actinomycetes can be horizontally acquired through male dispersal and sampling of actinomycetes from the soil [7,14].

The identities of the antifungals produced by attine ant-associated actinomycetes remain largely unknown. Only two compounds have been identified so far: a previously unknown antifungal named dentigerumycin that is produced by *Pseudonocardia *species isolated from the lower attines *Apterostigma dentigerum *and candicidin, a well known antifungal that is produced by *Streptomyces *species isolated from the higher attine ants belonging to the genus *Acromyrmex *[12,13]. *Pseudonocardia *isolated from *A. octospinosus *also inhibit the growth of *Escovopsis *in bioassays, but the antifungal compounds have not been isolated or identified [12].

The aims of this work were to isolate and identify actinomycete bacteria from *A. octospinosus*, identify antifungal compounds produced by these bacteria and thereby gain insights into whether the actinomycetes (i) co-evolved with the ants, as suggested by unusual antifungal compounds produced by *Pseudonocardia *mutualists, or (ii) were acquired from the environment, as suggested by the presence of well known antifungals that are widely produced by environmental isolates. We isolated actinomycetes from three colonies of *A. octospinosus *that were collected in Trinidad, identified two *Pseudonocardia *and nine *Streptomyces *species and chose single antifungal producing *Pseudonocardia *and *Streptomyces *species isolated from the same ant colony for further analysis. The *Streptomyces *species was found to produce candicidin and is closely related to the candicidin-producing *Streptomyces *bacteria isolated from *A. octospinosus *in Panama [12], supporting the hypothesis that candicidin-producing *Streptomyces *species are common mutualists of higher attines and are probably acquired via environmental sampling. The *Pseudonocardia *species produces an unusual antifungal compound that is related to the clinically important polyene antifungal nystatin. The isolation of these species suggests that the diversity of actinomycetes associated with attine ants probably occurs through both co-evolution of *Pseudonocardia *with the ants and environmental sampling.

This work also takes the total number of known antifungals associated with attine ants to three, two of which are associated with *A. octospinosus*, and provides the first direct biochemical evidence that a diversity of actinomycete symbionts translates into a diversity of antifungal compounds in attine ant colonies.

## Results

### Isolation and bioassay of actinomycetes

**Figure 1:**
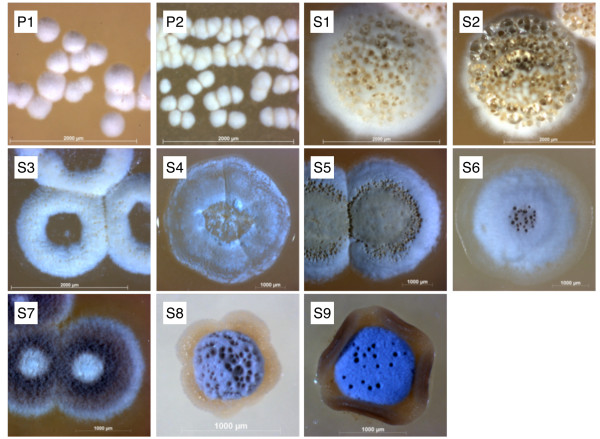
**Actinomycete species isolated from attine ants**. Actinomycete species isolated from *Acromyrmex octospinosus *worker ants viewed under a light microscope at 40 × magnification. *Streptomyces *strains are numbered S1-S9 and *Pseudonocardia *strains P1-P2.

**Figure 2:**
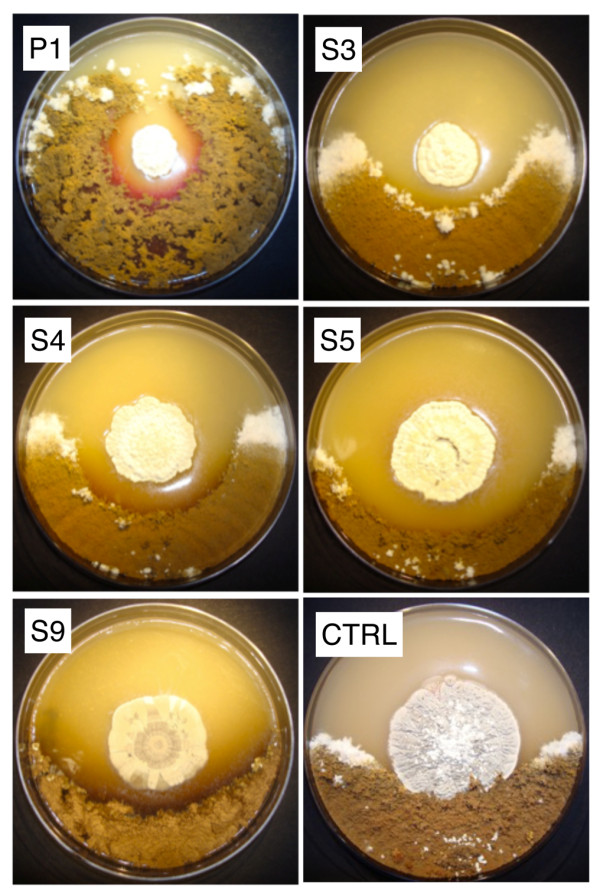
**Antifungal bioassays against *Escovopsis***. Bioassays against the fungal garden parasite *Escovopsis weberi*. The actinomycete strains S3, S4, S5, S9 and P1 formed clear inhibition zones while the control strain, *Streptomyes lividans*, produced no zone of inhibition and was overgrown by the nest parasite.

**Figure 3:**
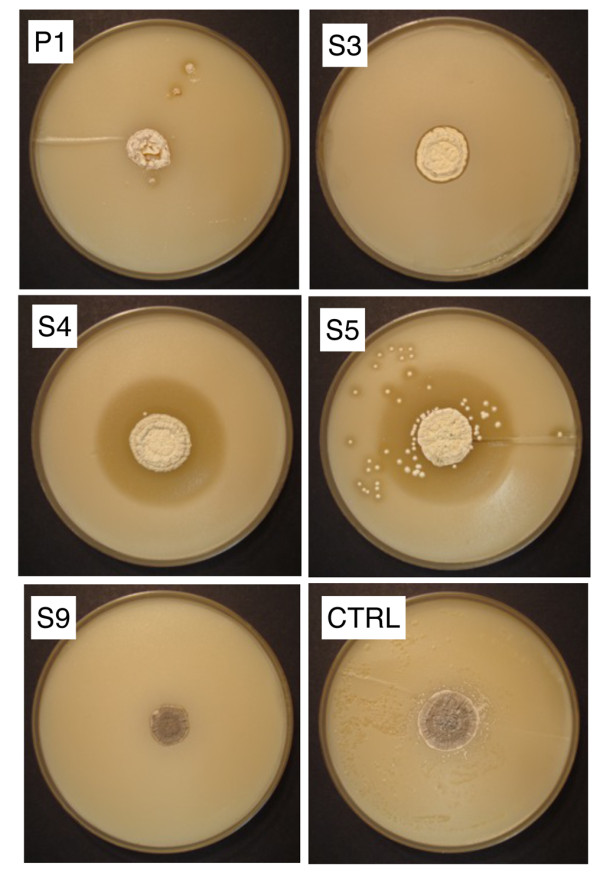
**Antifungal bioassays against *Candida***. Bioassays against the human pathogen *Candida albicans*. S4, S5 and, to a lesser extent, P1 all inhibit the growth of *C. albicans *whereas the control strain *Streptomyes lividans *is overgrown.

*A. octospinosus *ants from three colonies collected in Trinidad were either streaked directly onto HC and MS agar plates or washed in sterile water which was then spread onto the agar. Actinomycete colonies were purified by restreaking and then examined by light microscopy and identified by 16 S rDNA sequencing. Together with bacteria from other genera (*Tsukamurella *and *Nocardiopsis*) two *Pseudonocardia *(P1-P2) and nine *Streptomyces *(S1-S9) strains were isolated and identified (Figure 1, GenBank accession HM179225-HM179235). All bacterial strains were screened in bioassays against a strain of *Escovopsis weberi *isolated from an *A. octospinosus *nest and against *Candida albicans*, a human pathogen. Bioassays revealed that strains P1, S3, S4, S5 and S9 inhibit the growth of *E. weberi *when grown on MS agar (Figure 2) while P1, S3, S4 and S5 also inhibit the growth of *C. albicans *(Figure 3). The *Pseudonocardia *P1 strain has weak activity against *E. weberi *and very weak activity against *C. albicans *(Figures 2 and 3).

### *Streptomyces *S4 makes candicidin

A previous study revealed that a *Streptomyces *strain isolated from *A. octospinosus *in Panama makes the polyene antifungal candicidin [12] and a polymerase chain reaction (PCR) analysis of the nine *Streptomyces *and two *Pseudonocardia *strains using primers used by Haeder *et al*. in their study revealed that only *Streptomyces *S4 and S5 contain the candicidin biosynthesis genes *fscM *and *fscP *(Additional Files 1 and 2). Candicidin production was confirmed using liquid chromatography (LC) followed by tandem mass spectrometry (MS/MS) on butanol-extracted culture supernatants of *Streptomyces *S4 (Additional File 3). The *fscM *and *fscP *genes were not found in P1, S3, or S9, which suggests that they are producing antifungals not previously identified in the *A. octospinosus *mutualism. The PCR product amplified using *fscP *primers in the S9 sample was sequenced and is not *fscP*, consistent with its slightly larger size (Additional File 1).

### Genome scanning of *Pseudonocardia *P1

*Pseudonocardia *P1, isolated from the same ant colony as *Streptomyces *S4, produces a relatively small zone of inhibition in bioassays against *E. weberi *and a very small zone of inhibition against *C. albicans *(Figures 2 and 3). Furthermore, the antifungal activity of *Pseudonocardia *P1 was only detected on solid growth medium. This combination of factors made it difficult to purify sufficient antifungal compound(s) for analysis and identification. In order to gain further insight into the antifungal(s) produced by *Pseudonocardia *P1, we used 454-pyrosequencing to scan the genome of strain P1 (GenBank accession ADUJ00000000; Additional File 4). Analysis of the annotated contigs from this sequencing project revealed several polyketide synthase (PKS) gene fragments with > 90% amino acid identity to proteins involved in the biosynthesis of an antifungal compound named nystatin-like *Pseudonocardia *polyene (NPP) that is produced by *Pseudonocardia autotrophica *[15]. NPP is related to nystatin, a polyene antifungal that is made by *Streptomyces noursei *[16,17].

In order to determine whether or not *Pseudonocardia *P1 contains the entire biosynthetic gene cluster for a nystatin-like compound, contigs were aligned against the characterized NPP biosynthetic gene cluster from *P. autotrophica *(see Methods and Additional File 5). The tiled contigs spanned the entire cluster, including the six PKS genes that assemble the nystatin aglycone, the non-sugar containing backbone of nystatin. Full-length coding sequences were captured for 11 genes (*nypF, nypH, nypDIII, nypL, nypN, nypDII, nypDI, nypE, nypO, nypRIV, nypM*) that are proposed to be primarily involved in the post PKS-modification of the nystatin aglycone and two new genes, *nypY *and *nypZ*, with unknown functions (Table 1) [16]. Interestingly, a second glycosyltransferase, absent in *S. noursei *and *P. autotrophica*, is present in the *nyp *gene cluster and we have named it *nypY *(Table 1). The NypY protein belongs to the same glycosyltransferase family as NypDI, however it displays only 42% amino acid identity to NypDI and is therefore unlikely to be a functionally redundant copy of NypDI. This genome analysis strongly suggested that *Pseudonocardia *P1 has the genetic capacity to produce a nystatin-like polyene antifungal. PCR screening of the *Pseudonocardia *P2 strain and the nine *Streptomyces *strains isolated in this study suggests that none of them contain biosynthetic genes for a nystatin-like antifungal (Additional File 2).

**Table 1:** Nystatin P1 biosynthetic genes.

Contig ID	*Pseudonocardia *sp. P1 protein	Proposed function*	*P. autotrophica *ortholog	Identity (%)
PP100949	NypF	Phosphopantetheinyl transferase	NppF	89
PP100949	NypY	Glycosyltransferase	None†	--
PP100949	NypZ	Metallophosphoesterase	None‡	95
PP100398	NypH	ABC transporter	NppH	88
PP100398	NypDIII	dGDP-mannose-4,6-dehydratase	NppDIII	96
PP100400	NypL	P450 monooxygenase	NppL	84
PP100400	NypN	P450 monooxygenase	NppN	94
PP100400	NypDII	Aminotransferase	NppDII	96
PP100400	NypDI	Glycosyltransferase	NppDI	92
PP100821	NypE	Thioesterase	NppE	92
PP100306	NypO	Acyl-CoA decarboxylase	NppO	96
PP100306	NypRIV	LuxR transcriptional regulator	NppRIV	93
PP100306	NypM	Hypothetical protein	NppM§	82

*Proposed function of full length nystatin P1 biosynthetic (*nyp*) genes present in the draft genome of *Pseudonocardia *sp. P1 (Genbank accession ADUJ00000000).

† NypY is a glycosyltransferase unique to the nystatin P1 biosynthetic gene cluster and is not orthologous to proteins in the nystatin-like *Pseudonocardia *polyene (NPP) biosynthetic gene cluster from *P. autotrophica *(AC = EU108007) or the nystatin biosynthetic gene cluster from *Streptomyces nouresi *(AC = AF263912).

‡ The nystatin P1 and NPP biosynthetic gene clusters contain a putative metallophosphoesterase downstream of *nypH *and *nppH*, respectively that is not present in the nystatin biosynthetic gene cluster from *S. nouresi*. This open reading frame was not originally annotated by Kim *et al*. [15] and we have therefore given the *Pseudonocardia *P1 ortholog the name of *nypZ*.

§ *nyp*M encodes a hypothetical protein with high homology to NppM, which is annotated as a putative ferredoxin [15], however amino acid homology-based database searches failed to reveal homology to ferredoxin proteins.

### Identification of a nystatin-like compound in *Pseudonocardia *P1

**Figure 4:**
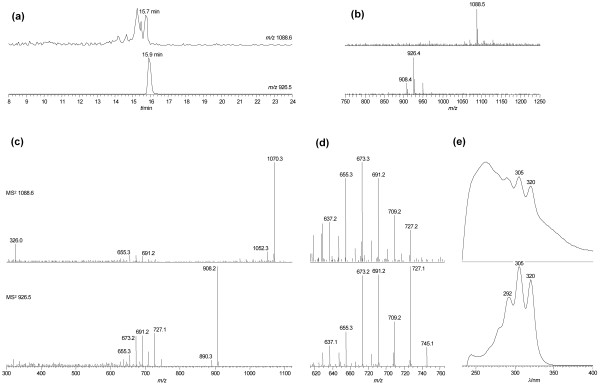
**Identification of nystatin P1**. Identification of a nystatin-like compound by liquid chromatography-tandem mass spectrometry. For each panel the lower tier corresponds to the nystatin standard and the upper tier to the *Pseudonocardia *P1 extract. (a) Extracted ion chromatograms for *m/z *926.5 (nystatin standard) and *m/z *1088.6 (nystatin P1). (b) Mass spectra averaged across the retention times indicated in panel A. (c) MS^2 ^analysis of the molecular ions identified above. The main mode of fragmentation is the loss of water molecules (*m/z *18). (d) Enlarged region of the MS^2 ^spectra. These product ions arise from loss of the carbohydrate portion plus one to seven water molecules and most are found in the nystatin standard as well as in nystatin P1. (e) Ultraviolet spectra extracted at the retention times indicated in panel A.

In order to determine whether *Pseudonocardia *P1 produces a nystatin-like antifungal compound, extracts of *Pseudonocardia *P1 were analysed by LC-MS/MS and compared to a nystatin A_1 _standard (Figure 4). Molecular ions for nystatin A_1 _(*m/z *926.5) or for NPP (*m/z *1129.6), produced by *P. autotrophica *[15] were not detected. However, a compound with a similar retention time on high-performance liquid chromatography (HPLC) to nystatin A_1 _and with a molecular ion of *m/z *1088.6 was identified (Figure 4a and b). This compound clearly, though somewhat concealed by the absorption of co-eluting peaks, shows the characteristic polyene absorption bands in its ultraviolet spectrum (absorption maxima at 292, 305 and 320 nm, Figure 4e). Together with the presence of nystatin-like biosynthetic genes in *Pseudonocardia *P1, the LC-MS/MS results strongly suggested that the P1-derived extract contained a nystatin-like compound. We have tentatively named this compound nystatin P1.

The mass difference of 162 observed between nystatin P1 and nystatin A_1 _suggested that nystatin P1 contains an additional hexose molecule. MS/MS fragmentation of the nystatin P1 ion (*m/z *1088.6) resulted in a series of product ions that are very similar to those derived from nystatin A_1 _(Figure 4c). All of the fragment ions corresponding to the nystatin P1 aglycone have corresponding counterparts in the nystatin A_1 _standard (Figure 4d). These data strongly suggested that the aglycone (backbone) of nystatin A_1 _and nystatin P1 is the same. Interestingly, the product ion with *m/z *326 is consistent with a mycosamine-hexose disaccharide and was only observed for nystatin P1. Further fragmentation of the *m/z *326 ion species corroborated the disaccharide nature of this moiety (Additional File 6).

The exact identity of the sugar molecules is, of course, speculative. Mycosamine is a probable component of nystatin P1 because this aminosugar is found in nystatin A_1 _and all the necessary genes for its biosynthesis and attachment to the aglycone have been identified in *Pseudonocardia *P1 (Table 1). Glucose is frequently found as a substituent in bacterial natural products. However, other natural hexoses such as mannose or galactose are also good candidates for the second sugar substituent. The attachment of the hexose to give nystatin P1 is most likely to be executed by the glycosyltransferase NypY (see above). The presence of the disaccharide in MS/MS furthermore suggested that the nystatin P1 aglycone is substituted at one position with a mycosamine-hexose moiety rather than the two sugar molecules being attached at separate positions.

## Discussion

We isolated actinomycetes from *A. octospinosus *garden worker ants and, in a single colony of ants, identified a *Pseudonocardia *and a *Streptomyces *species that produce antifungals in laboratory culture. The *Streptomyces *species, which we named S4, contains candicidin biosynthesis genes (Additional Files 1 and 2) and produces candicidin (Additional File 3), consistent with a report on antifungal-producing actinomycetes associated with *A. octospinosus *[12]. The actinomycetes studied in this work were isolated from *A. octospinosus *ants collected in Trinidad, whereas the previous study used *A. octospinosus *ants collected in Panama [12]. However, despite this geographic separation, the candicidin-producing *Streptomyces *strains identified in the two studies show 99% 16 S rDNA sequence identity suggesting that candicidin-producing *Streptomyces *are common mutualists of *A. octospinosus*. Candicidin-producing *Streptomyces *are widespread in the environment [18] and attine ants most likely acquire them selectively from the soil.

The *Pseudonocardia *species P1, isolated from the same colony as *Streptomyces *S4, showed relatively weak antifungal activity that was only observed in cultures grown on solid growth medium. This made it difficult to purify enough of the compound for analysis and identification. Using a genome scanning approach we identified a biosynthetic gene cluster for a polyene antifungal in *Pseudonocardia *P1 and then isolated and identified this antifungal using LC-MS/MS. This combined chemical and genomic approach provides a powerful tool for identifying and isolating new antibiotics and confirmed that *Pseudonocardia *P1 produces a polyene antifungal that we have tentatively named nystatin P1. This compound is markedly different from the antifungal dentigerumycin produced by *Pseudonocardia *associated with the lower attine ant species *A. dentigerum *[13] although it is notable that both *Pseudonocardia *strains are making previously unknown antifungals, consistent with the idea that the *Pseudonocardia *mutualists co-evolved with attine ants. We did not detect any compounds in extracts from *Pseudonocardia *P1 agar plates and mycelium that matched the isotopic mass of dentigerumycin. However, since the biosynthetic gene cluster for this compound is not known, we cannot exclude the possibility that this strain also has the ability to make dentigerumycin.

Taken together, this work provides the first direct evidence that individual leaf-cutting ant colonies have access to multiple antifungals via the diversity of hosted actinomycetes and increases the number of known antifungals used by attine ants to three. This work also provides evidence to support the two current possibilities for the identity and acquisition of mutualistic bacteria, *Pseudonocardia *co-evolution, and the environmental acquisition of useful actinomycetes. This strongly suggests that both possibilities apply, at least in the attine species *A. octospinosus*. Careful experimental work will be needed in order to demonstrate that multiple compounds are in fact produced and confer benefits *in vivo *[19]. It is interesting that the only two antifungal compounds to be isolated and identified from *A. octospinosus *colonies so far are polyenes, which are active against dimorphic fungi, yeasts (*Candida*) and molds (*Escovopsis*), but which apparently do not kill the fungal cultivar [12]. The isolation of a nystatin-like polyene from a leaf-cutting ant-associated *Pseudonocardia *species in this work agrees with the report by Sen *et al*. [11] that some *Pseudonocardia *bacteria associated with attine ants have non-specific antibiotic properties that inhibit a range of fungi and are not targeted specifically at *Escovopsis *[11].

The advantage to the ants of deploying two antifungals is not clear. Polyene antifungals are thought to work by interacting hydrophobically with ergosterol in the fungal cell membrane and forming channels that increase membrane permeability [20], but this may not be their only mechanism of action [21], and there may therefore be some advantage to the ants in using more than one. However, as fungi do not develop resistance to polyene antifungals (at least in a clinical setting), it is unlikely that resistance is the basis for any such advantage. Nevertheless, as candicidin and nystatin are not antibacterial, neither of these compounds is likely to be involved in competition amongst the bacteria for host resources. Thus, the identities of these two antifungal compounds are consistent with the longstanding hypothesis that these actinomycete associates of leaf-cutting ants can be mutualists of the ant and the attine fungus, provided that the compounds are applied correctly by the ant [11].

## Conclusions

We used a combined genomic and chemical approach that has proven useful for the identification of a new antifungal associated with *Acromyrmex *ants, this time produced by their *Pseudonocardia *mutualist. This approach should stimulate further chemical ecology studies of insect fungiculture systems, which are widespread in nature and which are likely to use symbiotic antibiotic-producing bacteria to protect their fungal partners [1]. We also provide evidence that supports both of the possibilities proposed to explain the mutualism between actinomycetes and attine ants-co-evolution of *Pseudonocardia *with attine ants and environmental sampling by the ants of useful antibiotic-producing bacteria. We propose that these possibilities are not mutually exclusive and that both are likely to apply to both attine ants and other systems of insect fungiculture.

## Methods

### Bacterial isolation and identification

Ants from three *A. octospinosus *colonies collected in Trinidad and Tobago were streaked onto hydrolysed chitin (HC) and mannitol plus soya flour (MS) agar plates [22,23] containing the antifungals nystatin and cycloheximide at final concentrations of 0.05 mg/mL. The remainder of the ants were washed in sterile water which was then spread onto HC and MS agar plates. Actinomycete isolates were colony purified and stored in 20% glycerol at -20°C. Genomic DNA was isolated from actinomycetes as described [23].

### 16 S rDNA analysis

A 1000 bp fragment of the 16 S ribosomal DNA gene was PCR-amplified using the following primers: 533F 5'-GTGCCAGCMGCCGCGGTAA-3' [24] and 1492R 5'-GGTTACCTTGTTACGACTT-3' [25]. The resulting PCR products were gel purified, sequenced (The Genome Analysis Centre, http://www.tgac.bbsrc.ac.uk/) and subsequently used to query the Green Genes database http://greengenes.lbl.gov/cgi-bin/nph-simrank_interface.cgi.

### Bioassays against *Escovopsis *and *Candida*

Spores (50 μL) of each actinomycete were inoculated into 10 mL liquid TSB/YEME (1:1) [23] and grown on a shaker (260 rpm, 30°C) for three days in order to generate mycelium. The mycelium was collected by centrifugation and resuspended in fresh TSB/YEME to yield a concentrated cell paste. The centre of an MS plate was inoculated with either 10 μL sterile TSB/YEME (negative control) or 10 μL of the concentrated cell paste and incubated for 10 days at 22°C, at which point the edge of the plate was inoculated with a small amount of mycelium of *Escovopsis weberi *(CBS 110660). The *Escovopsis *strain used in this study was obtained from CBS Fungal Biodiversity Centre http://www.cbs.knaw.nl and maintained on MS agar containing carbenicillin and streptomycin each at final concentrations of 0.05 mg/mL. Alternatively, *C. albicans *was inoculated into soft (0.5%) Lysogeny Broth agar, which was then was used to overlay the plate containing the actinomycete.

### 454-pyrosequencing and analysis

Genomic DNA was quantified using the Quant-it dsDNA HS Assay Kit (Invitrogen, CA, USA) and measured on a Qubit fluorometer (Invitrogen). An aliquot of 5 μg was used to generate the single stranded library for 454 pyrosequencing using the GS Titanium General Library Prep Kit according to the manufacturer's protocol (Roche, Hertfordshire, UK) except that, rather than fragmenting by nebulization, DNA was fragmented in a 100 μL volume using the Covaris-S2 ultra sonicator (K Biosciences, PA, USA) with the following settings-Mode: Frequency Sweep, Duty Cycle: 5%, Intensity: 3, Cycle Burst: 200 for two continuous cycles of 45 s. Library quality and quantity was assessed by running 1 μL of the library on a RNA PICO 6000 labchip (Agilent, CA, USA) and an emPCR titration was used to determine the optimal number of molecules per bead required to achieve the targeted 8% enrichment for the full scale emPCR. Approximately 790,000 enriched templated beads were subjected to 454 pyrosequencing on a quarter of a picotitre plate on the GS FLX sequencer (Roche) using the GS FLX Titanium Chemistry. The sequence reads were quality filtered and assembled into contigs using the Newbler Assembly v2 software (Roche).

Contigs were annotated using the Rapid Annotation Seed Technology Server [26]. Coding sequences annotated as polyketide synthases were extracted and inspected further by BlastP analysis against the National Center for Biotechnology Information non-redundant protein database, as well as Pfam [27] and non-ribosomal peptide synthetases-PKS [28]. NUCmer [29] using an 80% cutoff and the show-tiling utility were used to tile contigs to the *Pseudonocardia autotrophica *biosynthetic gene cluster for NPP [15]. Microsoft Excel was used to convert the output of the NUCmer show-tiling utility to Gene Finder Format and visualized using Artemis (release 11.22) [30].

### LC-MS analysis

The residue obtained from butanol-extracted *Streptomyces *S4 cultures (50 mL) grown in liquid MS was redissolved in 50% aqueous methanol (0.3 mL). The samples were centrifuged at maximum speed prior to injection (5 μL) into a Shimadzu single quadrupole LCMS-2010A mass spectrometer equipped with Prominence HPLC system. Compounds were separated on a Waters XBridge™ C18 3.5 μm 2.1 × 100 mm column using the following gradient (solvent A: 0.1 formic acid in water, solvent B: 0.1% formic acid in acetonitrile, flow rate 0.35 mL min^-1^): 0.01-0.5 min 15%B, 0.5-14 min 15-95%B, 14-16 min 95%B, 16-16.5 min 95-15%B, 16.5-19 min 15%B. Mass spectra were acquired in positive ion mode with the capillary voltage set to 1.3 kV.

A sporulating culture of the *Pseudonocardia *P1 isolate on MS agar was extracted twice with methanol (200 mL). The solvent was removed under reduced pressure and the residue redissolved in 50% aqueous methanol (150 μL). An authentic nystatin A_1_standard (Sigma-Aldrich, MO, USA) was prepared at 0.1 mg mL^-1 ^in 50% aqueous methanol. Immediately before LC-MS analysis, the crude extract and the standard were diluted twofold with 20% aqueous methanol and spun in a microcentrifuge at maximum speed for 4 min to remove any insoluble matter. Only the supernatant was used for injection (5 μL). The samples were run on a Surveyor HPLC system attached to a LCQ DecaXP^plus ^ion trap mass spectrometer (both Thermo Fisher, MA, USA). Separation was on a 100 × 2 mm 3 μ Luna C18(2) column (Phenomenex) with 0.1% formic acid in water as solvent A and methanol as solvent B using the following gradient: 0-20 min 20-95% B, 20-22 min 95% B, 22-23 min 95-20% B, 23-30 min 20% B. The flow rate was set to 260 μL min^-1 ^and the column temperature was maintained at 30°C. Detection was by ultraviolet (full spectra from 200-600 nm) and by positive electrospray MS using spray chamber conditions of 350°C capillary temperature, 50 units sheath gas, five units auxiliary gas, and 5.2 kV spray voltage. Targeted MS^2 ^with S4 and P1 extracts was performed with 35% collision energy and an isolation width of *m/z *4.0.

## Abbreviations

HC: hydrolyzed chitin; HPLC: high-performance liquid chromatography; MS: mannitol plus soya flour; MS/MS: tandem mass spectrometry; NPP: nystatin-like *Pseudonocarda *polyene; PCR: polymerase chain reaction; PKS: polyketide synthase.

## Authors' contributions

JB carried out the bacterial isolation and identification. RFS and SG contributed equally to this work. RFS carried out the genome sequence analysis. SG carried out the chemical isolations and identification. DH and ND sequenced and assembled the genome. MJB, DWY, RJMG and MIH conceived the study, participated in its design and coordination and helped to draft the manuscript. All authors read and approved the final manuscript.

## Supplementary Material

Additional file 1 **Detecting candicidin biosynthesis genes using polymerase chain reaction (PCR)**. PCR analysis of antifungal producers using primers against candicin biosynthesis genes ***fscM ***and ***fscP***. Sequence identities to Haeder ***et al***. [12]: ***fscM ***gene, S4 = 100%, S5 = 99%; ***fscP ***gene: S4 = 98% and S5 = 98% Click here for file

Additional file 2 ***Streptomyces *and *Pseudonocardia *strains identified in this study**. The *Pseudonocardia *and *Streptomyces *strains isolated in this study are listed with the *Acromyrmex octospinosus *colony they were isolated from (1,2 or 3), the accession numbers for their 16 S ribosomal DNA (rDNA) sequences, the top National Center for Biotechnology Information Blast hits for each of their 16 S rDNA sequences and the percentage identity to these BLAST hits. Also noted are the results from polymerase chain reaction testing for the candicidin biosynthetic genes *fscM *and *fscP *using primers from a previous study [12] and the nystatin-like *Pseudonocardia *polyene biosynthetic gene *nppDIII *using the primer set RFS84 (CAGATCCGCTTCTACCAGG) and RFS85 (CGCACCGAGTGCATCTG). Click here for file

Additional file 3 **Liquid chromatography-tandem mass spectrometry (LC-MS/MS) identification of candicidin in S4 extracts**. Analysis of S4-derived extracts. Left panel (A), ultraviolet spectrum extracted at RT 8.3 min (see panel B) from the S4 extract. The absorption maxima match those previously reported for candicidin D [12]. Right panel (B), LC-MS analysis of S4 extract. Ion chromatograms extracted for the molecular ion of candicidin D (*m/z *1109.6) are shown. (C), MS2 analysis of the extracted ion *m/z *1109.6. The fragmentation pattern of the antifungal compound from *Streptomyces *S4 perfectly matched the fragmentation of candicidin as reported previously [12]. The ions highlighted in the Haeder *et al*. study [12] are labelled in a larger font. Click here for file

Additional file 4 **genome sequencing data for *Pseudonocardia *P1**. Summary of the *Pseuodonocardia *sp. P1 draft genome sequence output obtained by 454 pyrosequencing Click here for file

Additional file 5 **Identification of the nystatin P1 biosynthetic gene cluster**. Tiling of *Pseudonocardia *sp. P1 contigs (GenBank accession ADUJ00000000) to the NPP biosynthetic gene cluster from *P. autotrophica *(GenBank accession EU108007). *The negative value for PP100949 denotes that the contig extends 4517 bp beyond the nystatin-like *Pseudonocardia *polyene biosynthetic gene cluster. **Negative values indicate that adjacent contigs overlap. Click here for file

Additional file 6 **MS^3 ^data for nystatin P1**. The spectrum shows the fragmentation data of the *m/z *1088 → 326 ion. The most frequently observed fragmentation corresponds to loss of water: *m/z *308 (-1 H_2_0), *m/z *290 (-2 H_2_0), *m/z *272 (-1 H_2_0). The *m/z *146 product ion is consistent with a mycosamine sugar after loss of the hexose (mass difference 180). Click here for file
