# Supplementary figures and images for: A mixed community of actinomycetes produce multiple antibiotics for the fungus farming ant Acromyrmex octospinosus
Source: BMC Biol. 2010 Aug 26;8:109. doi: 10.1186/1741-7007-8-109 (PMC2942817; doi:10.1186/1741-7007-8-109)

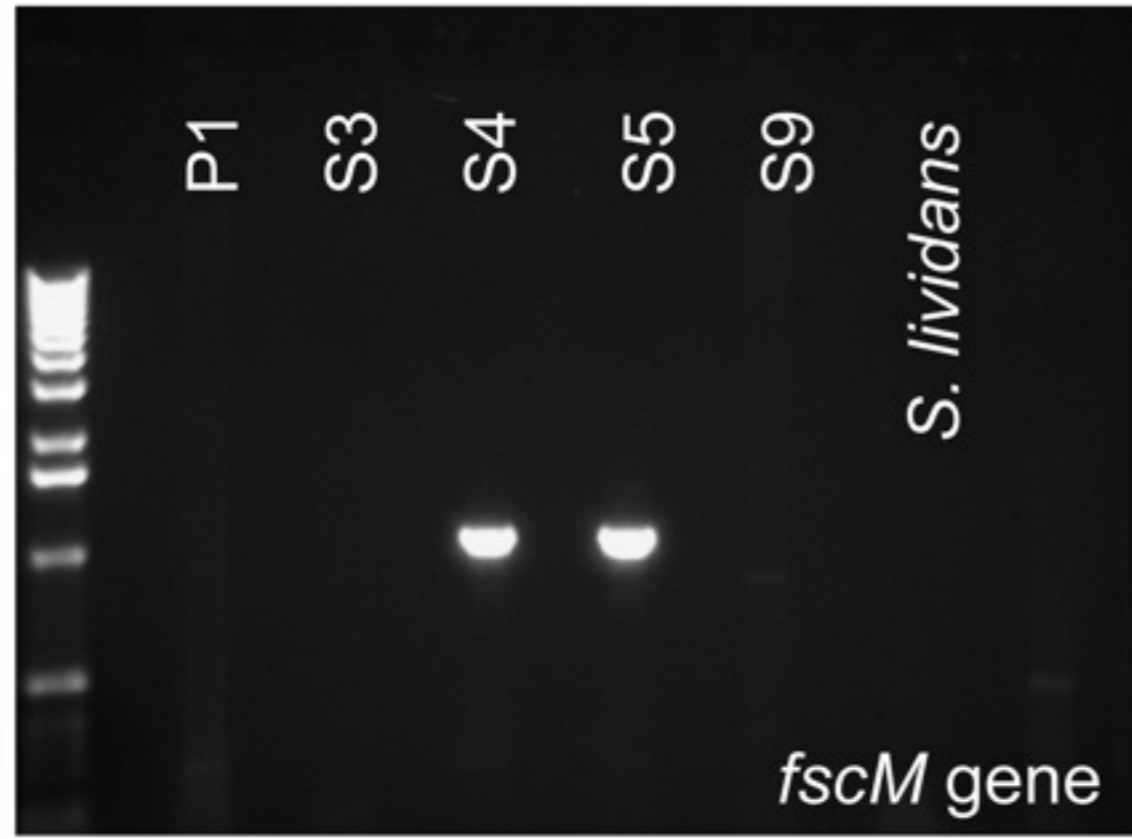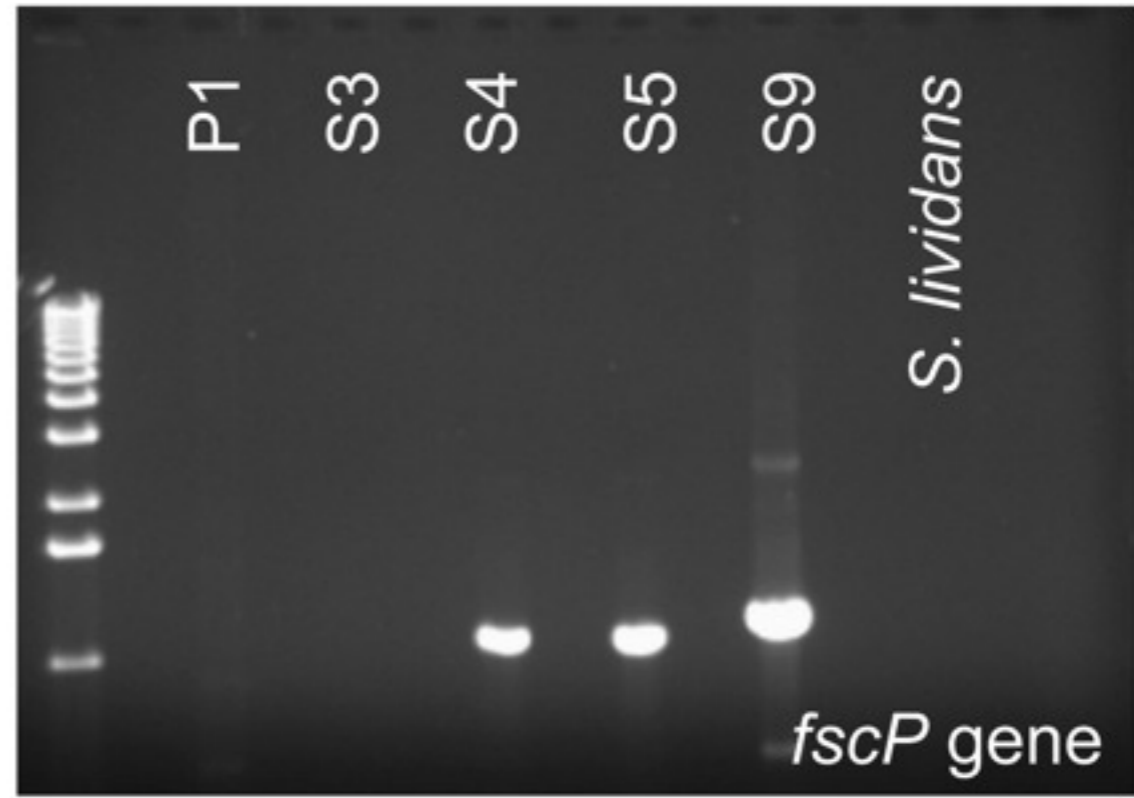

Supplement: Additional file 1 — Detecting candicidin biosynthesis genes using polymerase chain reaction (PCR). PCR analysis of antifungal producers using primers against candicin biosynthesis genes fscM and fscP. Sequence identities to Haeder et al. [12]: fscM gene, S4 = 100%, S5 = 99%; fscP gene: S4 = 98% and S5 = 98% [file 1741-7007-8-109-S1.PDF]

**A**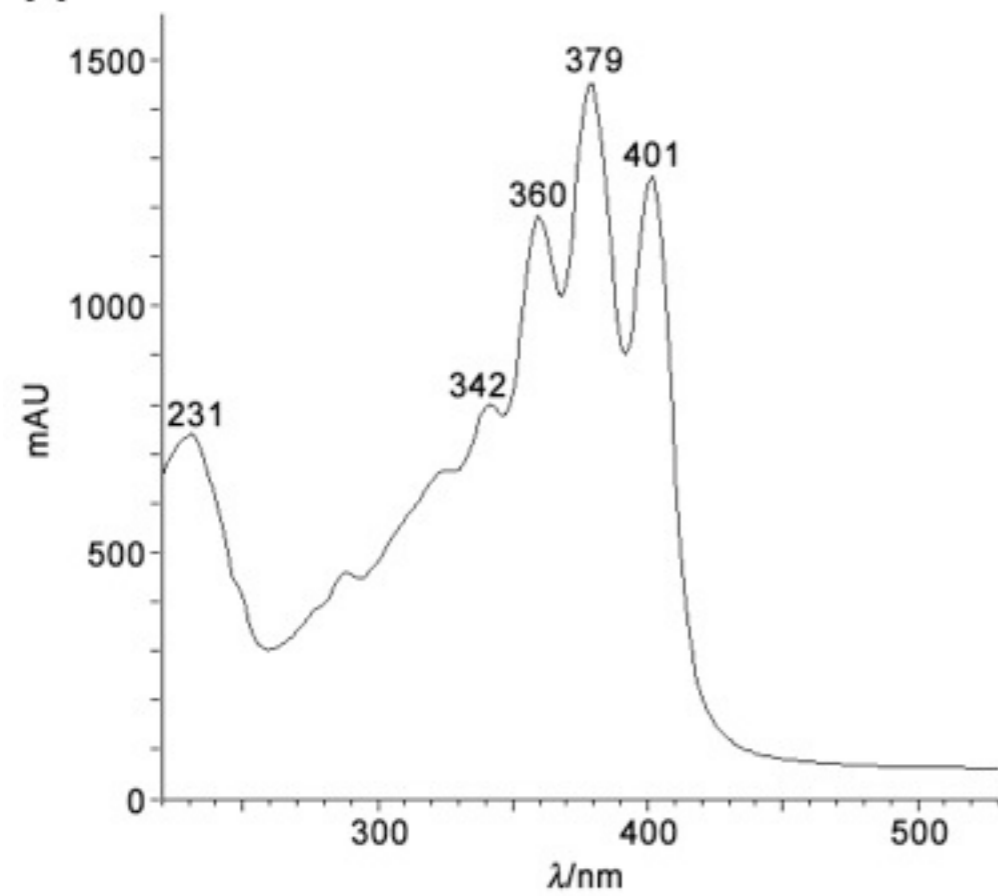**B**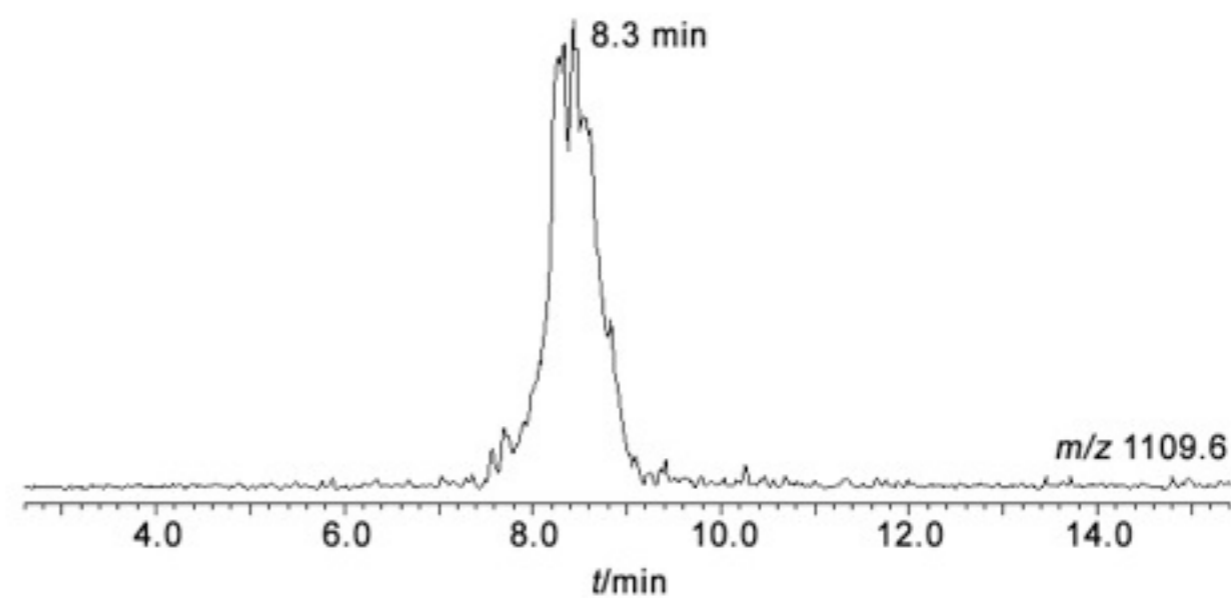

C

22a05 #1000-1004 RT: 17.55-17.61 AV: 2 NL: 3.51E7  
T: + c d Full ms2 1109.52@35.00 [295.00-2000.00]

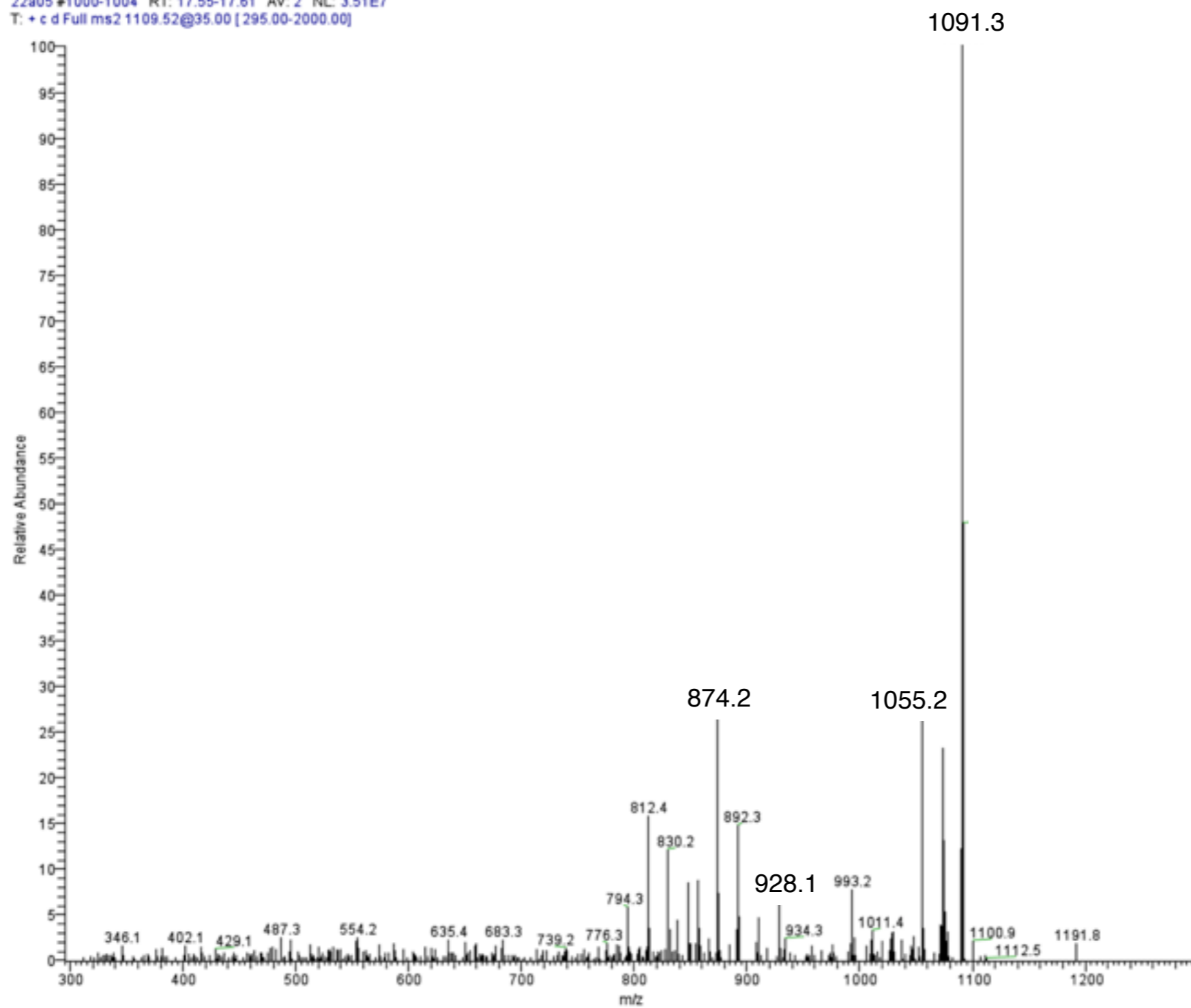

Supplement: Additional file 3 — Liquid chromatography-tandem mass spectrometry (LC-MS/MS) identification of candicidin in S4 extracts. Analysis of S4-derived extracts. Left panel (A), ultraviolet spectrum extracted at RT 8.3 min (see panel B) from the S4 extract. The absorption maxima match those previously reported for candicidin D [12]. Right panel (B), LC-MS analysis of S4 extract. Ion chromatograms extracted for the molecular ion of candicidin D (m/z 1109.6) are shown. (C), MS2 analysis of the extracted ion m/z 1109.6. The fragmentation pattern of the antifungal compound from Streptomyces S4 perfectly matched the fragmentation of candicidin as reported previously [12]. The ions highlighted in the Haeder et al. study [12] are labelled in a larger font. [file 1741-7007-8-109-S3.PDF]

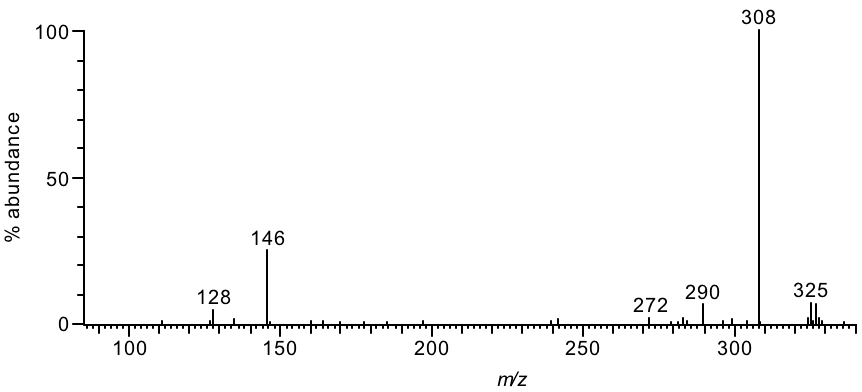

Supplement: Additional file 6 — MS3 data for nystatin P1. The spectrum shows the fragmentation data of the m/z 1088 → 326 ion. The most frequently observed fragmentation corresponds to loss of water: m/z 308 (-1 H20), m/z 290 (-2 H20), m/z 272 (-1 H20). The m/z 146 product ion is consistent with a mycosamine sugar after loss of the hexose (mass difference 180). [file 1741-7007-8-109-S6.TIFF]
