# Supplementary material for: A mixed community of actinomycetes produce multiple antibiotics for the fungus farming ant Acromyrmex octospinosus
Source: BMC Biol. 2010 Aug 26;8:109. doi: 10.1186/1741-7007-8-109 (PMC2942817; doi:10.1186/1741-7007-8-109)
Supplement: Additional file 2 — Streptomyces and Pseudonocardia strains identified in this study. The Pseudonocardia and Streptomyces strains isolated in this study are listed with the Acromyrmex octospinosus colony they were isolated from (1,2 or 3), the accession numbers for their 16 S ribosomal DNA (rDNA) sequences, the top National Center for Biotechnology Information Blast hits for each of their 16 S rDNA sequences and the percentage identity to these BLAST hits. Also noted are the results from polymerase chain reaction testing for the candicidin biosynthetic genes fscM and fscP using primers from a previous study [12] and the nystatin-like Pseudonocardia polyene biosynthetic gene nppDIII using the primer set RFS84 (CAGATCCGCTTCTACCAGG) and RFS85 (CGCACCGAGTGCATCTG). [file 1741-7007-8-109-S2.PDF]

**Strain name**	**Colony no.**	**GenBank accession**	**Sequence length (bp)**	**NCBI top blast hit**	**Description**	**Query coverage**	**Candicidin *fscM* gene (1035bp)**	**Candicidin *fscP* gene (1039bp)**	**Nystatin gene *nppDIII* (407bp)**
P1	3	HM179227	707	EU139568.2	*Pseudonocardia* sp. AL050512-17 16S ribosomal RNA gene, partial sequence	100%	-	-	+
P2	2	HM179232	825	EU139574.2	*Pseudonocardia* sp. CC031209-02 16S ribosomal RNA gene, partial	100%	-	-	-
S1	3	HM179225	750	HM579818.1	*Streptomyces* sp. HY27(2010) 16S ribosomal RNA gene, partial sequence	100%	-	-	-
S2	1	HM179226	708	HM579818.1	*Streptomyces* sp. HY27(2010) 16S ribosomal RNA gene, partial sequence	100%	-	-	-
S3	3	HM179228	725	HM235471.1	*Streptomyces* sp. 3bA 16S ribosomal RNA gene, partial sequence	100%	-	-	-
S4	3	HM179229	830	HM579798.1	*Streptomyces* sp. HY7(2010) 16S ribosomal RNA gene, partial sequence	100%	+	+	-
S5	2	HM179230	760	HM579798.1	*Streptomyces* sp. HY7(2010) 16S ribosomal RNA gene, partial sequence	100%	+	+	-
S6	3	HM179231	697	GQ924551.1	*Streptomyces* sp. ACT-0117 16S ribosomal RNA gene, partial sequence	100%	-	-	-
S7	1	HM179233	780	AB184101.2	*Streptomyces* collinus subsp. albescens gene for 16S rRNA, partial	100%	-	-	-
S8	3	HM179234	656	GQ863928.1	*Streptomyces* sp. HB320 16S ribosomal RNA gene, partial	100%	-	-	-
S9	3	HM179235	783	FJ190555.1	*Streptomyces* sp. MP9D2 16S ribosomal RNA gene, partial sequence	100%	-	-	-
