# Supplementary material for: A mixed community of actinomycetes produce multiple antibiotics for the fungus farming ant Acromyrmex octospinosus
Source: BMC Biol. 2010 Aug 26;8:109. doi: 10.1186/1741-7007-8-109 (PMC2942817; doi:10.1186/1741-7007-8-109)
Supplement: Additional file 4 — genome sequencing data for Pseudonocardia P1. Summary of the Pseuodonocardia sp. P1 draft genome sequence output obtained by 454 pyrosequencing [file 1741-7007-8-109-S4.PDF]

Sequence characteristics	
Number of reads	234,256
Mean length of reads (nt)	409
Total length of reads (nt)	95,794,824
Number of assembled contigs	975
Mean length of contigs (nt)	7,301
Length of longest contig (nt)	113,082
Total length of contigs (nt)	6,423,827
Calculated genome coverage	14.9
