# Supplementary material for: A mixed community of actinomycetes produce multiple antibiotics for the fungus farming ant Acromyrmex octospinosus
Source: BMC Biol. 2010 Aug 26;8:109. doi: 10.1186/1741-7007-8-109 (PMC2942817; doi:10.1186/1741-7007-8-109)
Supplement: Additional file 5 — Identification of the nystatin P1 biosynthetic gene cluster. Tiling of Pseudonocardia sp. P1 contigs (GenBank accession ADUJ00000000) to the NPP biosynthetic gene cluster from P. autotrophica (GenBank accession EU108007). *The negative value for PP100949 denotes that the contig extends 4517 bp beyond the nystatin-like Pseudonocardia polyene biosynthetic gene cluster. **Negative values indicate that adjacent contigs overlap. [file 1741-7007-8-109-S5.PDF]

Contig name	Contig length	Start position of NPP cluster*	End position of NPP cluster	Distance until next contig**	Alignment coverage (%)	Average identity (%)	Stand
PP100949	7730	-4517	3212	113	41	90	+
PP100398	6038	3326	9363	2085	98	92	+
PP100824	1794	11449	13242	60	100	91	+
PP100152	575	13303	13877	6	100	88	-
PP100883	1372	13884	15255	224	99	91	+
PP100195	2383	15480	17862	86	100	92	-
PP100810	729	17949	18677	67	90	93	+
PP100313	4009	18745	22753	62	100	91	-
PP100885	984	22816	23799	1593	100	90	+
PP100160	3380	25393	28772	1112	100	92	+
PP100596	3822	29885	33706	887	100	92	-
PP100881	2140	34594	36733	391	99	93	+
PP100884	1733	37125	38857	213	100	92	+
PP100221	2062	39071	41132	239	100	91	+
PP100209	1553	41372	42924	2954	90	91	-
PP100552	3055	45879	48933	2091	100	90	-
PP100088	3023	51025	54047	409	100	92	+
PP100702	1239	54457	55695	-241	82	90	+
PP100400	8390	55455	63844	542	91	92	-
PP100683	859	64387	65245	782	100	91	-
PP100828	3584	66028	69611	240	100	91	+
PP100291	2226	69852	72077	414	100	91	-
PP100512	1112	72492	73603	879	100	93	+
PP100670	542	74483	75024	225	100	92	-
PP100028	3568	75250	78817	2033	100	93	-
PP100163	1679	80851	82529	190	100	89	+
PP100862	1219	82720	83938	1238	85	90	-
PP100733	602	85177	85778	165	99	88	+
PP100175	1874	85944	87817	-6	99	90	-
PP100148	4174	87812	91985	248	100	88	+
PP100918	1878	92234	94111	0	100	92	-
PP100816	1175	94112	95286	1086	100	87	-
PP100564	797	96373	97169	196	99	89	-
PP100817	1792	97366	99157	160	100	91	+
PP100115	892	99318	100209	1792	99	90	-
PP100895	4521	102002	106522	298	100	90	+
PP100677	666	106821	107486	496	100	88	-
PP100821	1918	107983	109900	754	100	87	+
PP100535	1059	110655	111713	3595	100	92	+
PP100607	624	115309	115932	1560	100	87	+
PP100306	3523	117493	121015	911	89	89	-
PP100046	1401	121927	123327	2390	91	84	-
